# Docetaxel-Mediated Uptake and Retention of Gold Nanoparticles in Tumor Cells and in Cancer-Associated Fibroblasts

**DOI:** 10.3390/cancers13133157

**Published:** 2021-06-24

**Authors:** Abdulaziz Alhussan, Kyle Bromma, Monica Mesa Perez, Wayne Beckham, Abraham S Alexander, Perry L Howard, Devika B Chithrani

**Affiliations:** 1 Department of Physics and Astronomy, University of Victoria, Victoria, BC V8P 5C2, Canada; alhussan@uvic.ca (A.A.); kbromma@uvic.ca (K.B.); WBeckham@bccancer.bc.ca (W.B.); 2 Department of Biochemistry and Microbiology, University of Victoria, Victoria, BC V8P 5C2, Canada; monikm@uvic.ca (M.M.P.); phoward@uvic.ca (P.L.H.); 3 Radiation Oncology, British Columbia Cancer-Victoria, Victoria, BC V8R 6V5, Canada; AAlexander3@bccancer.bc.ca; 4 Centre for Advanced Materials and Related Technologies, Department of Chemistry, University of Victoria, Victoria, BC V8P 5C2, Canada; 5 Centre for Biomedical Research, Department of Biology, University of Victoria, Victoria, BC V8P 5C2, Canada; 6 Department of Medical Sciences, University of Victoria, Victoria, BC V8P 5C2, Canada; 7 Department of Computer Science, Mathematics, Physics and Statistics, Okanagan Campus, University of British Columbia, Kelowna, BC V1V 1V7, Canada

**Keywords:** gold nanoparticles, tumor cells, cancer-associated fibroblasts, docetaxel, uptake, retention, nanomedicine

## Abstract

**Simple Summary:**

Currently, radiotherapy and chemotherapy are the most commonly used options, in addition to surgery, to treat cancer. There has been tremendous progress in interfacing nanotechnology to current cancer therapeutic protocols. For example, nanoparticles are used as drug carriers in chemotherapy and as radiation dose enhancers in radiotherapy. However, most of the work to date has been focused on tumor cells. To make significant progress in this field, we need to consider the tumor microenvironment, especially cancer-associated fibroblast cells that promote tumor growth. Our study shows the potential of targeting both tumor cells and cancer-associated fibroblasts to reap the full benefits of cancer nanomedicine.

**Abstract:**

Due to recent advances in nanotechnology, the application of nanoparticles (NPs) in cancer therapy has become a leading area in cancer research. Despite the importance of cancer-associated fibroblasts (CAFs) in creating an optimal niche for tumor cells to grow extensively, most of the work has been focused on tumor cells. Therefore, to effectively use NPs for therapeutic purposes, it is important to elucidate the extent of NP uptake and retention in tumor cells and CAFs. Three tumor cell lines and three CAF cell lines were studied using gold NPs (GNPs) as a model NP system. We found a seven-fold increase in NP uptake in CAFs compared to tumor cells. The retention percentage of NPs was three-fold higher in tumor cells as compared to CAFs. Furthermore, NP uptake and retention were significantly enhanced using a 50 nM concentration of docetaxel (DTX). NP uptake was improved by a factor of three in tumor cells and a factor of two in CAFs, while the retention of NPs was two-fold higher in tumor cells compared to CAFs, 72 h post-treatment with DTX. However, the quantity of NPs in CAFs was still three-fold higher compared to tumor cells. Our quantitative data were supported by qualitative imaging data. We believe that targeting of NPs in the presence of DTX is a very promising approach to accumulate a higher percentage of NPs and maintain a longer retention in both tumor cells and CAFs for achieving the full therapeutic potential of cancer nanotechnology.

## 1. Introduction

Radiotherapy (RT), chemotherapy, and surgery are the most widely used approaches to treat cancer. RT is an essential element of curative treatment for many cancers, including breast, prostate, cervix, head and neck, lung, and brain. The major limitation to attaining a curative RT dose in high-risk (locally advanced) non-metastatic tumors is the high susceptibility of normal tissues to damage from radiation. Currently, we are nearing the limit of the RT dose given to patients, which creates a need for novel methods that enhance the effective dose to the tumor, while mitigating side effects. Moreover, one of the main problems in chemotherapy is that only ~0.1% of injected dose gets to the tumor when a free drug is used. Enhancing the targeted delivery of radiotherapy by incorporating NPs with high atomic number material such as gold has tremendous potential to maximize the radiation dose given to the tumor and minimize doses delivered to normal tissue [1,2,3,4,5]. Similarly, the use of NPs has shown promising results in overcoming some chemotherapeutic issues, where an increase of up to 5% of the injected chemotherapeutic dose within the tumor was observed [6]. Therefore, recent advances in nanotechnology can be exploited to overcome challenges in current cancer therapies.

**Figure 1:**
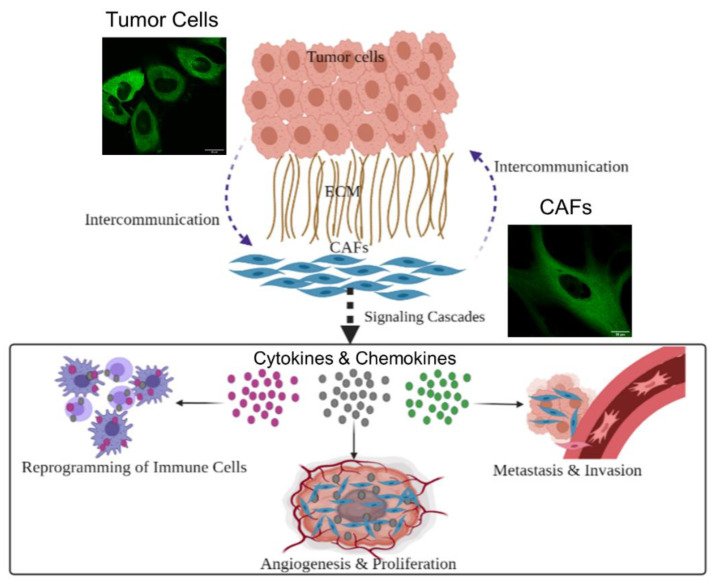
Schematic showing the role CAFs in tumor progression. The relationship between cancer cells and CAFs is mutual. Cancer cells recruit normal cells, turning them into CAFs to support the development of the tumor. On the other hand, CAFs secret several growth factors, enzymes and cytokines that promote the proliferation of the tumor, help with metastasis of tumor cells, and weaken the impact of the immune system on tumor cells. This intercommunication between cancer cells and CAFs fosters the ideal niche for tumor to develop. It is also worth mentioning that tumor cells are considerably smaller in size compared to CAFs, as illustrated in the two confocal images. Scale bar = 20 μm.

To design successful novel therapeutic approaches, it is very important to recognize the role of the tumor microenvironment (TME) in cancer development. The TME is composed of cells, such as normal fibroblasts, cancer-associated fibroblasts (CAFs), endothelial cells, pericytes, macrophages, lymphocytes, and other immune cells, as well as an acellular compartment comprising the extracellular matrix (ECM) and soluble factors [7]. Cells within the TME interact with each other and with cancer cells. Such tumor-stroma crosstalk could result in alliances to promote or suppress cancer growth. One very important interaction is between normal fibroblasts and cancer cells, where cancer cells transform normal fibroblasts into CAFs to facilitate tumor growth [8]. Normal fibroblasts are activated by cancer cells and can be identified by certain markers, such as fibroblast activation protein alpha and alpha smooth muscle actin [9,10,11,12]. Once activated, they play an integral role in cancer progression, as explained in the schematic in Figure 1. They promote tumor growth and proliferation by multiple pathways, including the recruitment of stromal cells, the modification of the ECM by secreting ECM remodeling enzymes, and encouraging angiogenesis by the recruitment of endothelial cells [8]. CAFs can also enable cancer invasiveness by creating routes in the stroma for cancer cells to travel through to other sites, and by secretion of host-derived cytokines, chemokines, and inflammatory mediators that promote growth and attenuate apoptosis [8]. Moreover, CAFs facilitate metastasis by producing TGF-β signaling, which encourages cancerous cells colonization of distant organs [9]. In addition, CAFs contributes significantly towards the immunomodulation of tumor tissue by releasing pro-inflammatory cytokines that recruit certain immune cells to the tumor stroma. Those cells then differentiate into tumor-associated immune cells and release several important endothelial and growth factors that promote the occurrence and progression of tumors. These factors contribute to chemotherapeutic resistance and engage in immunosuppression activities [8]. Thus, as CAFs play many roles in maintaining an optimal TME, they are gaining considerable attention from the scientific community. Their significant roles in cancer progression have made CAFs an important molecular target for the treatment of cancer [13]. A successful approach in cancer therapy should include co-targeting of cancer cells and vital components of their microenvironment, particularly CAFs. This could potentially reduce tumor growth and proliferation, lessen tumor invasion, inhibit metastatic dissemination, and disturb the tumor-associated immune response [14]. Targeting CAFs through their markers requires addressing the variation in CAF markers between different cancer types and different CAFs subpopulation, which poses a huge challenge. Considering the important role of CAFs in tumor progression, we focused principally on a simpler way to target tumor cells and CAFs in our current study.

According to previous studies, targeting of only cancer cells is not sufficient to obtain the full benefits of in cancer treatments [14]. As most studies have mainly focused on tumor cells, our study is designed to elucidate the NP interactions in tumor cells and in CAFs. We used gold nanoparticles (GNPs) as our model NP system, since they can be used as a radiation dose enhancer in radiotherapy and a drug carrier in chemotherapy [15,16,17,18,19]. The biocompatibility of GNPs has been determined in a phase I clinical trial [20]. In order to target these GNPs, a peptide sequence which includes an integrin-binding domain, RGD was used since there is an over-expression of the integrin-binding domain in tumor cells and CAFs [21]. Recent studies have shown the promise of combining a commonly used chemotherapeutic: DTX with GNPs [21]. DTX stabilizes microtubules within the cells, arresting them in the most radiosensitive phases of the cell cycle, G2/M [19,21]. Furthermore, the presence of DTX results in an increase in the accumulation of NPs within tumor cells, making it very promising in both chemotherapy and radiotherapy. However, we lack data on the extent of DTX’s effect on CAFs of different tumor origins. In this study, we used three cancer cell lines (MIA PaCa-2 (pancreatic), PC3 (prostate), and HeLa (cervical)) and three CAF cell lines (CAF-98 (pancreatic), CAF-D6006T (prostate), and Hs 895.T (melanoma)). A previous study has compared differences between NP behavior in a cervical cancer cell line (HeLa) and a CAF cell line from melanoma origin [21]. However, in this study, we added CAFs and cancer cells of similar origins (prostate and pancreatic) along with the two cell lines used for the abovementioned previous study for comparison.

Hence, our study will address the following unanswered questions:

(1) Is there a difference in NP uptake between tumor cells and CAFs?
(2) What is the ability of tumor cells and CAFs to retain NP?
(3) Can we improve the NP uptake in both tumor cells and CAFs using DTX? How significant is the effect in both tumor cells and CAFs?
(4) Can we significantly improve the retention of NPs in tumor cells and in CAFs using DTX?
(5) Is there a significant difference in the NP behavior in tumor cells and in CAFs based on our study?

## 2. Materials and Methods

### 2.1. Gold Nanoparticle Synthesis

GNPs of 10.9 ± 1.2 nm diameter were produced using a citrate reduction method. Sodium citrate tribasic dihydrate (HOC(COONa)(CH_2_COONa)_2_·2H_2_O) was used as the reducing agent. A 1% solution of the reducing agent was made for the GNP synthesis process. Tetrachloroauric(III) acid trihydrate (AuCl_4_H·3H_2_O) was used to make a 1% gold salt solution. To synthesize GNPs, 900 µL of the 1% gold solution was added to 90 mL of double-distilled water in an Erlenmeyer flask and was stirred and heated until boiling. When starting to boil, 1800 µL of the 1% reducing agent was added rapidly and was stirred while boiling for 10 min. The color of the solution changed to a crimson red during this time to indicate the creation of GNPs. The heat was then turned off, and the solution was stirred at room temperature for 10 more minutes.

### 2.2. Gold Nanoparticles Functionalization

Polyethylene glycol (PEG) was used at a surface density of 1 PEG per nm^2^ of the GNP surface area (835 PEG per GNP) to functionalize the negatively charged GNPs to create GNP_PEG_. To improve the uptake of GNP_PEG_, a peptide containing RGD integrin-binding domain was added at one molecule of RGD for every two PEG molecules to create GNP_PEG–RGD_. For live cell confocal imaging, 2000 Da PEG and 3400 Da PEG-thiol-CY5 were used in equivalent amounts, adding up to 835 PEG per GNP and 1 RGD molecule per PEG molecule to create GNP_PEG–CY5–RGD_ (fluorescent CY5 dye; ~651 nm excitation, ~670 nm emission).

### 2.3. Gold Nanoparticles Characterization

Perkin Elmer λ 365 ultraviolet visible (UV-VIS) spectrophotometer, Anton Paar LiteSizer 500 dynamic light scattering (DLS) and ζ potential were used to determine the shape, size, concentration, hydrodynamic radius, and surface charge, of GNPs, GNP_PEG_, and GNP_PEG–RGD_, respectively. GNPs shape and size were verified using Transmission Electron Microscopy (TEM) (Ultra-high Resolution Scanning Electron Microscope SU9000, Hitachi, Pleasanton, CA. USA).

### 2.4. Cell Culture Methodology

Human pancreatic cancer cell line MIA PaCa-2 (ATCC#: CRL-1420™), prostate cancer cell line PC-3 (ATCC#: CRL-1435™), cervix cancer cell line HeLa (ATCC#: CCL-2™), and melanoma cancer-associated fibroblasts (CAFs) Hs 895.T (ATCC#: CRL-7637™) were obtained from the American Type Culture Collection. Human pancreatic cancer-associated fibroblasts (CAF-98) were derived from resected PDAC tumor tissue from a consenting patient through the Gastrointestinal (GI) Biobank at the Vancouver General Hospital. Prostate cancer-associated fibroblasts (CAF-D6006T) were provided by the Vancouver Prostate Centre (VPC). All cells were cultured in high glucose Dulbecco’s modified Eagle medium (DMEM; Gibco, ThermoFisher Scientific, Waltham, MA, USA) enriched with 10% fetal bovine serum (FBS; Gibco), 1% penicillin/streptomycin (Gibco), and 4 mM of GlutaMax (Gibco). For cell detachment and cell fixations, trypsin–EDTA(Gibco) and paraformaldehyde (PFA; Sigma Aldrich, Oakville, ON, Canada) were used, respectively. Phosphate-Buffered Saline (PBS) was used for cell washing and cell incubations occurred at 37 °C with 5% CO_2_.

### 2.5. Image Preparation

Confocal microscopy (Zeiss LSM 980, Carl Zeiss Microscopy GmbH, Jena, Germany) was used to visualize GNP distribution in cells. Live cells were imaged using oil-immersion 60X lens. Cells were cultured on 35 mm coverslip-bottom dishes (MatTek, Ashland, MA USA) with 2 mL of media and incubated for 24 h. All cells were dosed with 7.5 µg/mL of GNP_PEG-CY5-RGD_ post-incubation and the ones that required DTX treatment were treated with 50 nM of DTX before incubating for another 24 h. Microtubules cells were stained with CellLight™ Tubulin-GFP BacMam 2.0, ThermoFisher Scientific, Waltham, MA, USA), providing specific targeting to cell tubulins. The tubulin stainer was added to the cells for at least 16 h prior to imaging. Twenty-four hours after GNP treatment, the media of the uptake study samples was substituted with colorless media (FluoroBrite DMEM; Gibco, ThermoFisher Scientific, Waltham, MA, USA) and four drops of NucBlue^®^, ThermoFisher Scientific, Waltham, MA, USA) Live reagent (DAPI) (Hoechst^®^ 33,342 dye; ~350 nm excitation, ~461 nm emission, ThermoFisher Scientific, Waltham, MA, USA) was added to stain the nucleus of each cell. These samples were then incubated for 20 min before imaging. At the same time, the media of the retention study samples was replaced with fresh media and cells were incubated for 72 h. After this 72-h period, the media of these cells was changed with colorless media and DAPI dye was added to each sample, incubated for 20 min, then imaged. Imaging settings were kept as identical for all samples.

### 2.6. Quantification of Cellular Uptake and Retention

All cells were dosed with 7.5 µg/mL of GNP_PEG−RGD_. For each cell line, ~1 × 10^5^ cells were incubated in 6-well dishes in 3 mL of media. In total 12 wells were used for every cell line; 6 for the uptake study (3 treated with 50 nM DTX and 3 untreated with DTX) and 6 wells for the retention study (3 treated with 50 nM DTX and 3 untreated with DTX). All cells were placed in the incubator for 24 h. The uptake study cells were then washed three times with PBS, trypsinized, and incubated for 5 min for detachment. Meanwhile, the media of the retention study cells was replaced with fresh media and cells were incubated for an additional period of 24 h. After 72 h, the retention study cells were washed three times, trypsinized, and incubated for 5 min. For both the uptake and the retention studies, media was added to the cells, and they were carefully counted using a hemocytometer counting chamber and transferred to glass tubes for processing. Cells were then treated with aqua regia (3:1 molar ratio of HCl and HNO_3_ (VWR)) and heated in a mineral oil bath at 90 °C for ~30 min. For each tube, 100 μL of hydrogen peroxide (VWR) was added before being incubated in a mineral oil bath for ~30 min to ensure full consumption of all cell contents. Finally, the samples were diluted to 2.5% *v/v* (volume per volume) acid content with deionized water. Inductively Coupled Plasma–Mass Spectrometry (ICP-MS; Agilent 8800 Triple Quadrupole) was used to measure the gold content in every tube, providing the amount of gold in parts per billion (ppb) or ng/mL. The number of gold nanoparticles per cell was calculated using the following equation:

$$\begin{array}{c} \frac{Gold\;nanoparticle\;}{Cell}=\\ \frac{\frac{Gold\;Concentration}{Sample}\left[\frac{g}{\mathrm{mL}}\right]\times Sample\;Volume\;\left[\mathrm{mL}\right]\times N_{A}\left[\frac{\mathrm{atoms}}{\mathrm{mol}}\right]}{Gold\;atomic\;mass\left[\frac{g}{\mathrm{mol}}\right]\times \mathrm{Number}\;of\;Cells\times \frac{\;Gold\;atoms}{Gold\;nanoparticle}} \end{array}$$

where gold concentration per sample, the sample volume, and the number of cells per sample vary among our different samples, $the\;atomic\;mass\;of\;gold=196.96657\frac{g}{\mathrm{mol}},$ $N_{A}$ is Avogadro’s number ($6.022\times 10^{23}\;\frac{\mathrm{atoms}}{\mathrm{mol}}$), and the number of gold atoms per gold nanoparticle is calculated using the following equation:

$$\begin{array}{c} \frac{\;Gold\;atoms}{Gold\;nanoparticle}=\frac{Atoms\;per\;unit\;cell\times Gold\;Nanoparticle\;Volume\;\left[nm^{3}\right]}{Unit\;cell\;Volume\;\left[nm^{3}\right]}\\ =\frac{4\times \frac{4\pi r^{3}}{3}}{a^{3}}=\frac{2}{3}\pi {\left(\frac{D}{a}\right)}^{3} \end{array}$$

where *D* = 10.9 nm, which is the core diameter of a spherical gold nanoparticle, and *a* = 0.408 nm is the length of a unit cell. Gold synthesized by the citrate reduction method develops a face-centered cubic crystal structure with four atoms of gold contained in each unit cell. It is assumed that the distribution of nanoparticles in each cell type is even, and it is also assumed that the size of GNPs is homogenous; hence, the calculations only represent a group average. These two assumptions were informed by the images obtained from confocal for GNPs’ distribution, and TEM for GNPs’ size.

### 2.7. Cell Cycle Analysis

For cell cycle analysis, cells were cultured in 60 mm dishes with 5 mL of media and each cell line was divided into four different groups: nontreated control cells, 6-h DTX -treated cells, 24-h DTX-treated cells, and 72-h post DTX-treated cells. The latter was incubated with DTX for 24 h before refreshing the media and incubating for 72 h. All DTX -treated cells were treated with 50 nM of DTX. Following their respective incubation periods, cells were trypsinized using Trypsin-EDTA and neutralized with 10%FBS/DMEM, before being transferred to 15 mL polystyrene tubes. After spinning at 350× *g* for 5 min at 4 °C, the supernatant was poured off and the cell pellets were washed with ~1 mL PBS/1.0 × 10^6^ cells. The cell pellet–PBS solutions were spun at 350× *g* for 5 min at 4 °C and the pellets were resuspended in 1% PFA (Paraformaldehyde in PBS). Cells were then incubated on ice in the dark for 15 min to complete the fixation process. They were then spun at 350× *g* for 5 min at 4 °C, washed in 1 mL of PBS, and spun again at 350× *g* for 5 min at 4 °C. The samples were resuspended in 70% ethanol before they were incubated at –20 °C for a couple of days for further processing and fixation. Post-incubation, the samples were centrifuged at 350× *g* for 10 min at 20 °C, washed in 0.5% BSA-Bovine Serum Albumin in PBS, and centrifuged at 350× *g* for 5 min at 20 °C. The samples were resuspended in PBTB (PBS, 0.5% BSA, 0.1 % Triton-X 100) and RNase A was added at a concentration of 100 ug/mL. This mixture was shaken at 37 °C for 25 min to allow for cell membrane permeabilization and RNA degradation. The samples were wrapped in aluminum foil and propidium iodide (fluorescent at 488 nm with emission centered at 600 nm) was added at a concentration of 10 µg/mL before incubating on a shaker at 4 °C for 60 min. Samples were then centrifuged at 350× *g* for 5 min at 20 °C to label DNA. This step is essential because the amount of DNA in the cell reveals the phase the cell is in. Samples were resuspended in 1 mL PBS/BSA and passed through 50 µm cell strainer, before being run on a flow cytometer (FACS Calibur, BD Biosciences, Franklin Lakes, NJ, USA).

## 3. Results and Discussion

### 3.1. Characterization of Gold Nanoparticles

**Figure 2:**
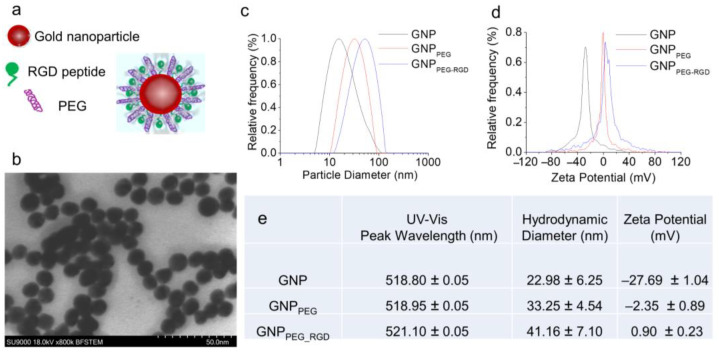
Characterization of GNPs. (**a**) Schematic diagram of GNP functionalized with both PEG polyether and RGD peptides to create the GNP_PEG-RGD_ complex used in this study. (**b**) TEM image of GNPs with a core size of 10.9 nm (± 1.2 nm). Scale bar is 50 nm. (**c**,**d**) Distributions of the hydrodynamic diameters, and ζ-potentials for pure GNPs, GNP_PEG_, and GNP_PEG-RGD_, respectively. GNP_PEG_ is GNP functionalized with PEG, which acts as a stabilizing agent; GNP_PEG-RGD_ is a GNP functionalized with both PEG and RGD, which enhances GNP uptake. (**e**) Table with the peak absorption wavelengths, hydrodynamic diameters, and mean ζ-potentials for pure GNPs, GNP_PEG_, and GNP_PEG-RGD_. Note: the error is represented by the standard deviation over three different measurements.

The goal of this study was to understand the difference in NP uptake and retention between cancer cells and CAFs for better integration of nanomedicine to current cancer therapy considering the role of CAFs in tumor growth. We used ~10 nm diameter GNPs, functionalized with both PEG and RGD peptide (Figure 2a), to compare the functionalized GNP uptake and retention for tumor cells and CAFs. Studies have shown that these smaller NPs penetrate better through tissues compared to larger NPs [22]. However, the uptake of smaller NPs at individual cell level is lower compared to larger NPs [23]. The cellular uptake of these smaller NPs can be increased significantly by adding a peptide containing integrin-binding domain, RGD [24,25,26,27,28]. The addition of the RGD peptide requires a stabilizing agent to avoid aggregation; although pentapeptide is most used for this purpose, we instead used polyethylene glycol (PEG) because an RGD/PEG combination (Figure 2a) allows better translation of this work to future in vivo studies and clinical trials. Based on transmission electron microscopy (TEM) imaging, the average core diameter of synthesized GNPs was 10.9 ± 1.2 nm (Figure 2b; Supplementary Figure S1). The hydrodynamic diameter of citrate capped as-made GNPs was 22.98 ± 6.25 nm (Figure 2c). It was increased to 41.16 ± 7.10 nm with the addition of the RGD peptide and PEG molecules (Figure 2c). The molecular weight of RGD-peptide and PEG were 1670 and 2000 Da, respectively. The addition of PEG and RGD peptide also resulted in the replacement of the negatively charged citrate molecules, which led to a significant change in the surface charge from negative (−27.69 ± 1.04 mV) to slightly positive (0.90 ± 0.23 mV) (Figure 2d). The peak wavelength of UV-visible absorption spectrum of bare GNPs was 518.8 nm and it is aligned with the peak wavelength for 10–15 nm GNPs (Supplementary Figure S2) [29]. There was only a slight red shift of the surface plasmon absorption peak wavelength for RGD/PEG-modified GNPs (GNP_PEG-RGD_), since both the RGD peptide and PEG molecules were considerably smaller. The GNP_PEG-RGD_ complex was used for this study to determine the GNP uptake and retention for different cancer cell lines and CAFs.

### 3.2. Cellular Uptake and Retention of GNP_PEG-RGD_ Complex

**Figure 3:**
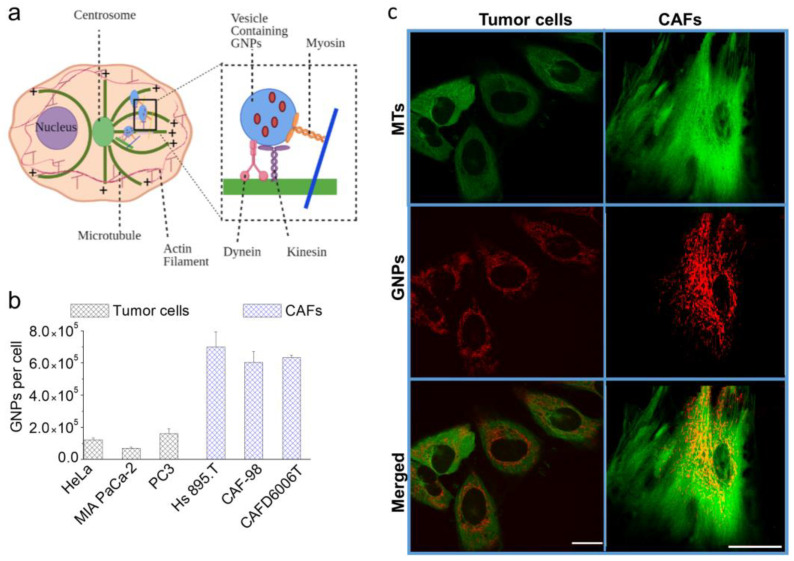
GNP Uptake and Intercellular Transportation. (**a**) Schematic illustration displaying the transportation of vesicles containing GNPs within the network of microtubules (MTs). MTs are hollow tubes composed of tubulin dimers constructed into a linear chain of protofilaments. They are polar structures that have a fast-growing “positive” end and a slow-growing “negative” end. MTs emerge from an organelle in the center of the cell known as a centrosome or microtubule organizing center (MTOC), with the positive ends always directed outwards in the process of nucleation. Inset figure: the motor proteins, dynein and kinesin, support the transportation of vesicles along the MT network. (**c**) Confocal microscope images of tumors cells vs. CAFs, showing MTs alone in green (**first row**), GNPs alone in red (**second row**), and both MTs in green and GNPs in red merged (**third row**). (**b**) GNP uptake by tumor cells and CAFs in the absence of DTX. Scale bar = 20 μm.

Receptor mediated endocytosis (RME) accounts for the majority of NP cellular uptake [30,31]. In this process, targeting ligands on the NP surface connect with cell surface receptors followed by internalization via endocytosis. Internalized NPs are first trapped in endosomes, which then fuse with lysosomes for further processing. Most receptors are recycled back to the cellular membrane, while vesicles containing processed NPs return to the cell periphery for excretion [32,33]. As illustrated in Figure 3a, microtubules (MTs) mediate the intracellular transport of NP-containing vesicles. MTs are long tubulin polymers and provide directional transport within the cell interior, while actin filaments support short-distance travel near the cell periphery [34,35]. NP transport along MTs is bidirectional and it is supported by two motor proteins: kinesin (transport toward the (+) end of MTs) and dynein (toward the (−) end) (see inset Figure 3a). Closer to the cell periphery, the myosin motor protein moves cargo along actin filaments (see inset Figure 3a). A map of the MT network and vesicles containing NPs in tumor cells and CAFs is given in Figure 3b (see also Supplementary Figures S3 and S4). Based on what we discussed so far, we believe that any disturbance to the function of MTs could have a significant impact on NP transport [36,37].

The extent of the cellular uptake of GNPs for tumor cells (HeLa (cervical), MIA PaCa-2 (pancreatic), PC3 (prostate)) and CAFs (Hs. 895T (melanoma), CAF-98 (pancreatic), CAFD6006T (prostate)) within a 24-h incubation period is plotted in Figure 3c. Our GNP uptake experiments were carried out at clinically feasible concentrations of 1.5 nM; as such, concentrations are more relevant in vivo. After a 24-h incubation, CAFs had a three-fold higher NP uptake in comparison to cancer cells (Figure 3c), which suggests that targeting CAFs, in addition to tumor cells, is feasible for better therapeutic outcomes. This might be attributed to the much larger size of CAFs compared to tumor cells coupled with the higher number of RGD binding integrins that CAFs express and their varieties easing the internalization of NPs [38]. Our results are consistent with a previous study that compared NP uptake between a cervical cancer tumor cell line (HeLa) and CAFs derived from melanoma [39]. We included these two cell lines as references and tested our approach on pairs of cells of pancreatic origin (MIA PaCa-2 and CAF-98)) and prostate origin (PC3 and CAFD6006T). We found no significant difference in GNP uptake among different CAFs, but there was a significant difference in GNP uptake among different tumor cell lines. The different in size between the used tumor cell lines and the higher degree of variation in the expression of integrins between the different tumor cells compared to CAFs may explain this result [40]. PC3 is larger in size compared to HeLa and MIA PaCa-2 and internalized the highest number of GNPs, whereas the latter two are comparable in size, but there still was a significant difference in their GNP uptake. All three tumor cell lines used, prostate, cervical, and pancreatic, express integrin ανβ3, which is able to recognize the RGD sequence conjugated on surface of GNPs. However, the cervical cell line also expresses another RGD binding integrin, ανβ6, which might explain the higher number of GNPs in them compared to MIA PaCa-2 [40].

**Figure 4:**
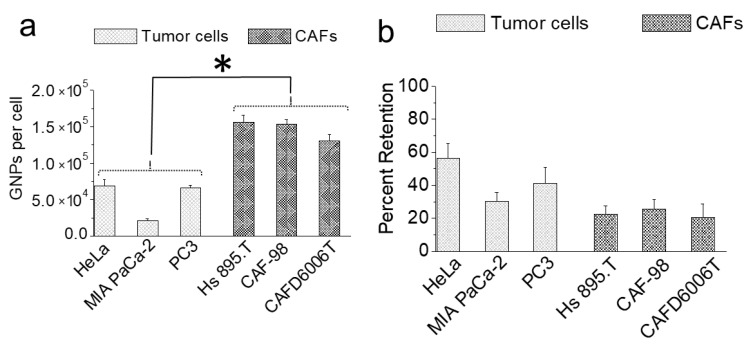
Retention of GNPs 72 h after introducing fresh media. * indicates *p* < 0.05. (**a**) GNPs retention in tumor cells vs. CAFs per cell. (**b**) The percent of GNPs retained by tumor cells and CAFs.

In a real TME, NPs are delivered to cells through tumor vasculature. Cellular retention of NPs over time is critical for delivering the optimum therapeutic dose. To simulate retention in this study, NPs were removed from the media after the cells were incubated with NPs over a 24-h period. The extent of NP retention between tumor cells and CAFs over a 72-h period is illustrated in Figure 4. We extrapolated the percent retention of NPs (Figure 4b) using the data in Figure 3c and Figure 4a. Exocytosis and the redistribution of NPs via cell division could explain the reduction in the cellular GNP content [32,33,41].

There was no significant difference in NP retention among different CAFs. This result is not surprising, considering that NP uptake was also very similar among different CAFs. As CAFs have a large doubling time, cell division likely had a less significant effect on NP retention compared to exocytosis over the 72-h incubation. For example, the number of NPs in CAFs was six times higher than in tumor cells for a 24-h incubation period (Figure 3c). The higher NP abundance in CAFs compared to tumor cells implies that, as soon as the NP-rich growth medium was replaced with fresh media, the CAFs were exposed to a higher concentration gradient compared to the tumor cells [42]. Therefore, the lower retention in CAFs compared to tumor cells could be explained by the dynamics of the exocytosis process. Percent retention of NPs in two tumor cell lines, HeLa and PC3, were three- and two-fold higher than CAFs, respectively. Among the three tumor cell lines, MIA PaCa-2 is the smallest in size and had the lowest retention. We believe that the NP concentration within MIA PaCa-2 was higher; however, they responded faster to the concentration gradient, resulting in a lower number of NPs within them at the end. Overall, the extent of the decrease in NP retention in tumor cells could be due to a combination of exocytosis dynamics and redistribution of NPs via cell division [43]. We found no correlation between the cell division time and the percent retention among the different tumor cells. For example, cell division times for PC3, HeLa, and MIA PaCa-2 are 28, 20, and 40 h, respectively. The percent retention in these tumor cells was roughly twice as low compared to our previous studies, which was expected because our previous studies measured retention after a shorter period of 24 h [32].

### 3.3. Modulation of NP Transport Using DTX

**Figure 5:**
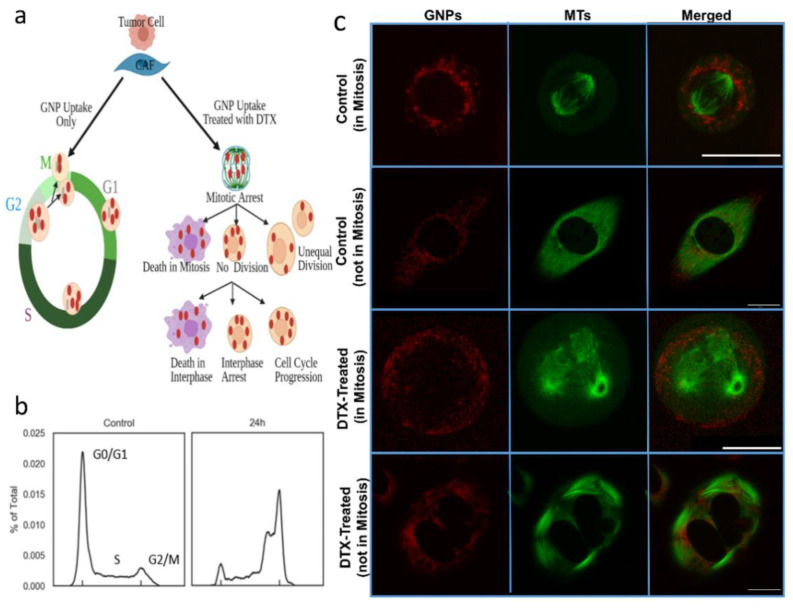
DTX effect on tumor cells and CAFs. (**a**) An illustration showing normal cell division (**left**) vs. the effect of DTX on cell division (**right**). (**b**) Cell cycle analysis between cells treated with 0 and 50 nM of DTX. (**c**) Confocal Images of control tumor cells and tumor cells treated with DTX. GNPs are in red (**first column**), MTs are in green (**second column**), and merged (**third column**). The first row shows the distribution of GNPs in control tumor cell in mitosis. The second row maps the vesicles containing GNPs and the MTs in a control cell not in mitosis (and not treated with DTX). The third row shows cell division in a tumor cell treated with DTX and stuck in mitosis. The fourth row shows the vesicles containing GNPs and the MTs in a cell treated with DTX and not in mitosis. Scale bar = 20 μm.

As explained before in Figure 3a, MTs facilitate the intracellular transport of vesicles containing NPs [33]. Therefore, modulating the MT network could drastically affect NP behavior within cells. In this study, we used the common, clinically approved anticancer drug DTX to stabilize MTs, which would ultimately affect internal NP transportation and their exocytosis from cells. Without DTX, cells undergo the regular cell cycle process by which cells reproduce normally going through the four major cell cycle phases (Figure 5a (left)); G1, S, G2, and M. The genetic information is replicated during the synthesis (S) phase and the cell divides into two daughter cells during mitosis (M). S phase and mitosis are separated by the Gap phases, G1 and G2 [42].

MTs can be modulated with antimitotic drugs such as DTX, which could lead to many different outcomes, as illustrated in Figure 5a (right). It has been shown that the cell type, antimitotic drug used, and its concentration has a combined effect in determining the fate of the cell [44]. As shown in Figure 5b, a higher fraction of cells treated with 50 nM DTX was arrested in the G2/M phase of the cell cycle compared to untreated cells. We were able to capture a control cell undergoing mitosis (Figure 5c (1st row)), which shows the normal formation of mitotic spindles out of microtubules, stretching between two ‘asters’ originating at centrosomes at either pole at mitosis. The DNA is arranged at a metaphase plate between the asters before the chromosomes are evenly separated into daughter cells. The second row in the image panel (Figure 5c (2nd row)) shows the map of vesicles containing GNPs and MTs in a control cell not in mitosis. With DTX treatment, the stabilization of MTs prevents spindle assembly during the mitosis phase of the cell cycle, and thus, prevents cell division [45,46]. Instead, multiple asters are formed, creating multiple cleavage planes (Figure 5c (3rd row)). This results in blocking of the cell cycle at the G2/M phase, as confirmed in Figure 5b (right). We also observed changes in the cell morphology in a cell not in mitosis (Figure 5c (4th row)) with the stabilization of MTs due to DTX treatment. These changes resulted in NPs getting trapped within the cell because of their inability to move along MTs. The next goal was to investigate how DTX affects the NP dynamics in tumor cells and in CAFs.

### 3.4. Determining the Effect of DTX on NP Uptake and Retention

As illustrated in Figure 3a, the major pathway of NPs entering the cell is via endocytosis [33]. Once NPs are internalized, their vesicular transport within the cell interior is along MTs. Considering the action of DTX on MTs, we expect a significant change in intracellular dynamics of internalized NPs. A previous study has reported a significant increase in the NP uptake with the treatment of DTX using a cervical cancer cell line, HeLa [47,48]. Therefore, we used HeLa as one of our reference tumor cell lines for comparison. However, it is not yet known how DTX affects NP uptake and transportation in CAFs, which play a major role in tumor progression. As DTX is a clinically approved drug that treats many cancers and acts as a complementary radiosensitizing agent in chemotherapy, understanding the role of DTX on GNP transportation could pave the way for a better chemoradiation approach with GNPs as a radiosensitizing agent [19]. Taxol-based anticancer drugs are used to treat prostate and pancreatic cancer [19]. Docetaxel is one of those taxol-based drugs. Therefore, we used both prostate and pancreatic tumor cells and CAFs for this study.

**Figure 6:**
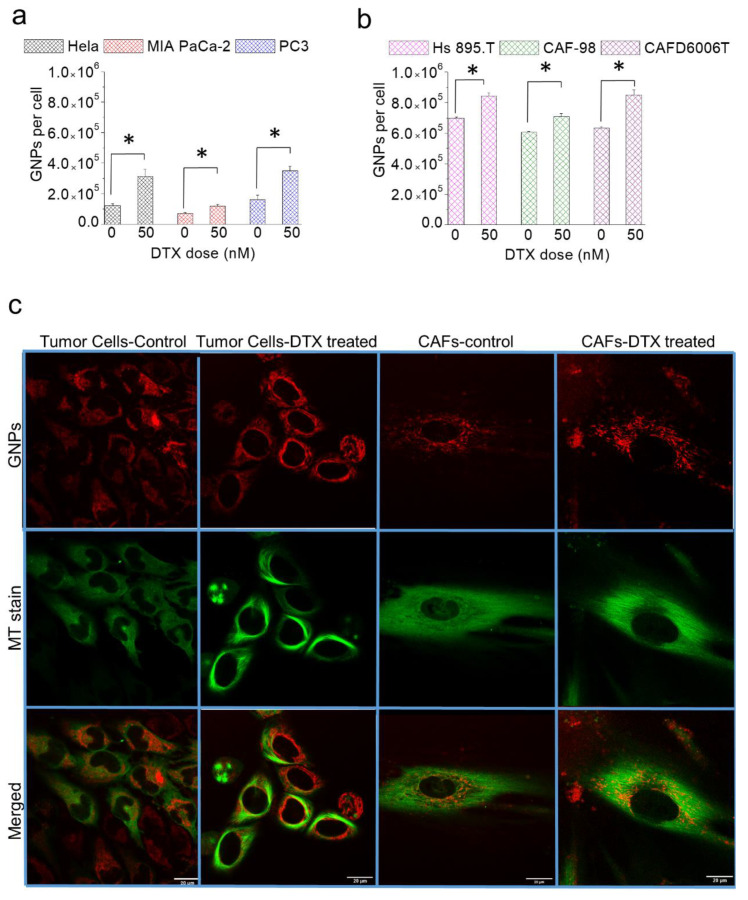
Effect of DTX on GNP. (**a**,**b**) The number of GNPs present in tumor cells and CAFs after a 24 h incubation with GNPs/DTX vs. GNPs alone, respectively. * indicates *p* < 0.05 (**c**) Confocal Images of tumor cells and CAFs treated and untreated with DTX. GNPs are in red (**first row**), MTs are in green (**second row**), and merged are in red and green (**third row**). The first column demonstrates the distribution of GNPs in cancer cells in the absence of DTX. The second column demonstrates the effect of DTX on MTs and on the distribution of GNPs in cancer cells. The third and fourth columns show the effect of the absence and presence of DTX on the number of GNPs in CAFs, respectively. Scale bar = 20 μm.

We used GNPs with a diameter of 10 nm for this study because the higher surface curvature of smaller NPs enabled efficient interaction between the RGD targeting ligand and cell surface integrins [48,49]. We used concentrations of 1.5 and 50 nM of GNPs and DTX, since such concentrations are also feasible in vivo, respectively [39,50]. A simultaneous incubation of both NPs and DTX over a 24-h period was used for the uptake study. As illustrated in Figure 6, the number of internalized GNPs in both tumor cells (Figure 6a) and CAFs (Figure 6b) increased dramatically for the simultaneous incubation vs. GNPs alone. The increase in GNP uptake in CAFs was significantly higher compared to tumor cells. The percent increase in NP accumulation in tumor cells was 155% (HeLa; cervical cancer), 70% (MIA PaCa-2; pancreatic cancer), and 115% (PC3; prostate cancer), while it was 20.6% (Hs 895.T; melanoma), 5.8% (CAF-98, pancreatic), and 34% (CAFD6006T; prostate) for CAFs. Based on the confocal images in Figure 6c (see also Supplementary Figures S3 and S4), DTX had a prominent effect on tumor cells as compared to CAFs. This is supported by cell cycle data (discussed in the next section), where we have a higher percentage of cancer cells arrested in the G2/M phase compared to CAFs. We speculate that one of the reasons for the lesser effect of DTX on CAFs is that since DTX works by directly binding to MTs and since CAFs are much larger than tumor cells, the effect of a small concentration of DTX used on CAFs’ larger cytoskeleton structure and more abundant MT network is more distributed, and thus, it has a weaker effect on CAFs compared to tumor cells. The progression of cell population toward G2/M was more dynamic in tumor cells compared to CAFs (see Supplementary Figure S5).

Based on the images, the distribution of GNPs was significantly changed in tumor cells in contrast to CAFs. It could be seen that GNP clusters were either localized closer to the nucleus or in areas where there were no MTs. However, the number of GNPs present in CAFs was still ~200% higher than in tumor cells with the treatment of DTX, even though the percent increase is lower than tumor cells. The increase in GNPs with the treatment of DTX can be attributed to two main reasons. Firstly, cells are arrested during mitosis (metaphase) following treatment with DTX. Therefore, the resulting prolonged time spent in the M phase could lead to increased NP accumulation. Secondly, we believe that DTX did not affect the endocytosis process, since it is largely mediated through the actin cytoskeleton closer to the cell membrane [50,51]. However, the stabilizing of MTs could affect the processing and removal of GNPs from cells, potentially trapping the NPs within cells and increasing their presence over time. This could lead to increase in accumulation intracellularly. Since MT stabilization could affect intracellular processing and removal of NPs from cells, we investigated how feasible it is for cells to retain NPs once we remove both GNPs and DTX from the cell culture media.

### 3.5. Intracellular Retention of GNPs in the Presence of DTX

One of the advantages of using GNPs in cancer nanomedicine is its dual ability to act as a drug carrier and a radiation dose enhancer [19]. The ability of cells to retain GNPs over an extended period of time would enable exploiting the full therapeutic potential in their applications in nanomedicine. Cellular transport and processing of NPs is dependent on MTs, and the use of MT-stabilizing drugs, such as DTX, could alter this process significantly, as mentioned before [34,35]. More specifically, DTX is expected to retard the NP removal from cells. This implies that DTX should increase in NP retention because of reduced exocytosis. In order to investigate the effects of DTX on NP retention, cells were first simultaneously incubated with NPs and DTX over a 24-h period. After the incubation period, the old media was replaced with fresh media (no GNPs or DTX), followed by an incubation period of 72 h to evaluate NP retention.

**Figure 7:**
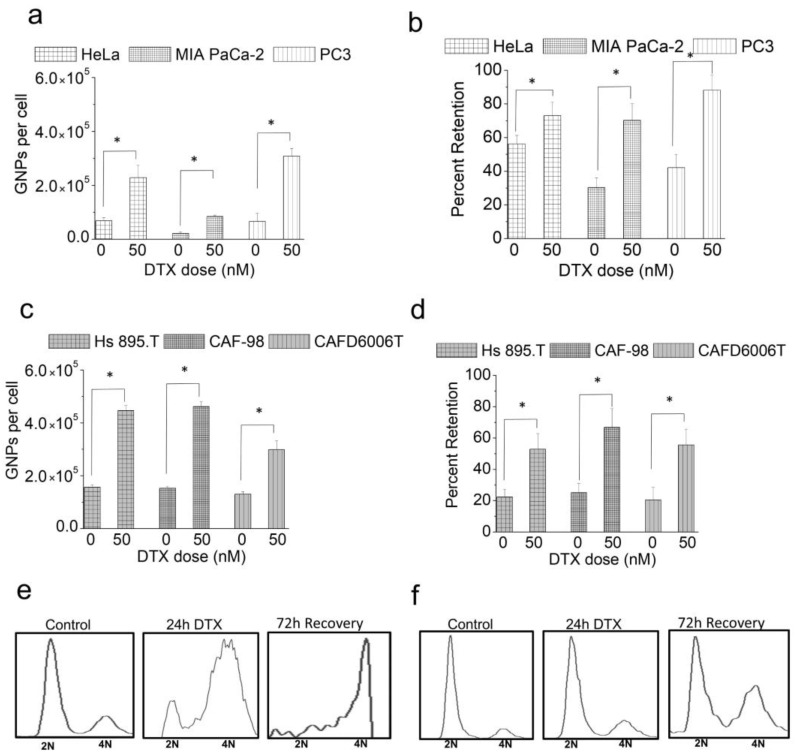
Retention of NPs in the presence of DTX. (**a**) Number of GNPs retained per cell for cancer cells that are untreated and treated with DTX. (**b**) Percent retention of GNPs for cancer cells that are untreated and treated with DTX. (**c**) Number of GNPs retained per cell for CAFs that are untreated and treated with DTX. (**d**) Percent retention of GNPs for CAFs that are untreated and treated with DTX. * indicates *p* < 0.05. (**e**,**f**) Cell cycle data, where 2N represent the cells in the G0/G1 phase and 4N represents the cells in the G2/M phase. MIA PaCa-2 (**e**) and CAF-98 that are (**f**) untreated, treated with DTX, and post recovery.

After a 72-h period, the tumor cells treated with GNPs alone had ~20% of their original NP content, while the ones treated with both GNPs plus DTX had 70% of their NP content (Figure 7a,b). A higher fraction of tumor cells was still in the G2/M phase even after 72 h. Therefore, this retardation of the cell division and the cells’ inability to process and transport NPs to the cell periphery for their excretion could have led to this observed increase in NP retention. The potential of NP retention was somewhat lower in CAFs compared to tumor cells, as illustrated in Figure 7c,d. The number of GNPs remaining in the CAFs was still more than 100% higher compared to tumor cells after 72 h (Figure 7c). However, CAF percent retention was lower compared to tumor cells, with values of 53%, 66.8%, and 55.6% for HS 895.T (melanoma), CAF-98 (pancreatic), and CAFD6006T (prostate), respectively. This fact implies that CAFs were able to excrete NPs at higher rate than tumor cells, lowering the retention. This could be explained with Figure 7e,f, where cell cycle data show a difference in the phase distribution of the cell populations of tumor cells (Figure 7e) and CAFs (Figure 7f). A larger population of tumor cells was still in the G2/M phase, which prevents NP removal via regular exocytosis, trapping GNPs for a longer time. In contrast, the population of CAFs arrested in G2/M phase was lower, which permitted more exocytosis of NPs and less retention, indicating a weaker effect of DTX on them.

**Figure 8:**
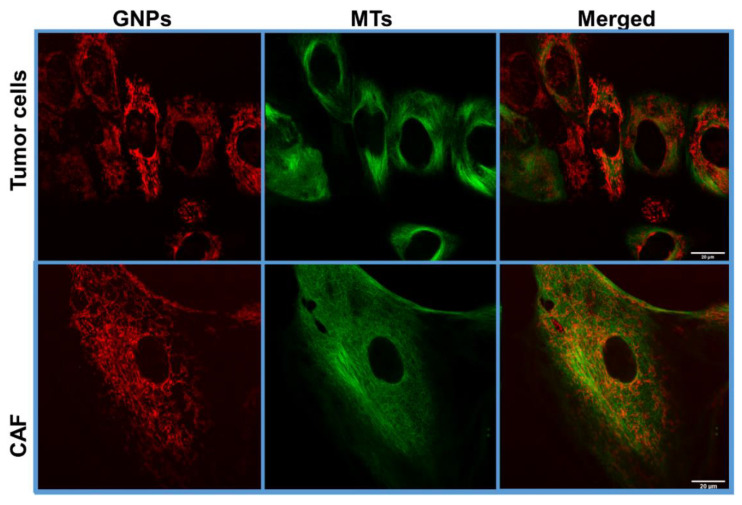
Retention of GNPs in tumor cells and CAFs post recovery. Confocal Images of tumor cells and CAFs 72 h post recovery. GNPs are in red (**first column**), MTs are in green (**second column**) and merged are in red and green (**third column**). The first row shows the distribution of GNPs in a group of tumor cells. The second row shows the presence of GNPs and the MTs in a CAF cell. Stabilization of MTs was present more in tumor cells compared to CAFs. Scale bar = 20 μm.

Moreover, more MT bundling was present in tumor cells compared to CAFs (Figure 8), which again implies a greater DTX effect on tumor cells compared to CAFs. The difference in the effect of DTX on tumor cells vs. CAFs is expected because different cell lines have different half maximal inhibitory concentration (IC50) for DTX. However, for the sake of mimicking a real-life scenario, we opted for using the same concentration of DTX for both tumor cells and CAFs. Nevertheless, if we compared untreated vs. treated cells, the increase of percent retention was higher for CAFs than for tumor cells, which indicates that the concentration of DTX used was indeed effective in increasing the retention rate of GNPs in CAFs, despite the lower percentage of cells arrested at the G2/M compared to tumor cells. It is also important to recognize that the number of GNPs present in CAFs was still much higher than that of tumor cells (Figure 7a,c and Figure 8). We believe that both the synchronization of tumor cells at the most radiosensitive phase, i.e., G2/M, and the higher number of radiosensitizing NPs concentrated in CAFs could be exploited in radiotherapy.

## 4. Conclusions

Incorporation of nanomedicine to current cancer modalities, such as chemotherapy, radiotherapy, and chemoradiation, would improve therapeutic outcomes while potentially reducing side effects. CAFs are indisputably involved in many phases of cancer development and are essential components of the ECM and stroma. Thus, they have much potential as therapeutic targets. Despite the interest in new technologies for cancer therapy, most efforts have solely focused on tumor cells instead of the whole TME, which alone is insufficient. It is important to deactivate or exterminate CAFs to control tumor growth. Our study sheds light on targeting not only tumor cells but also CAFs using NPs. We were able to show that the use of a clinically approved anticancer drug, DTX, further enhances the uptake and retention of NPs in both tumor cells and CAFs. The improvement of NP retention over an extended period of time allows for the delivery of therapeutics efficiently. Our approach is feasible in the clinic considering the clinically safe concentrations used in this study and the fact that DTX is given once a week to patients. Our study shows the potential of using even fewer GNP injections in fractionated radiotherapy considering the higher fraction of NPs retained with the use of DTX. The potential of GNPs to act as a radiosensitizer would ultimately allow us to reduce the radiation dose to further reduce the side effects. This type of novel approaches could ultimately improve patient care in the near future. Future studies would include exploring NPs uptake and retention in other important components of the TME in the presence and absence of DTX in vitro before transitioning to in vivo models. The potential of targeting two critical components of the TME, tumor cells and CAFs, with a single NP system will enable us to reap the full benefits of cancer nanomedicine to reach these goals.

## Data Availability

Datasets generated and/or analyzed during the current study are available from the corresponding author on reasonable request.

## Supplementary Materials

The following are available online at https://www.mdpi.com/article/10.3390/cancers13133157/s1, Figure S1: TEM images of GNPs; Figure S2: UV-Visible data; Figure S3.1: MTs and NPs in untreated HeLa; Figure S3.2: Change in MTs and NP distribution in HeLa treated with 50 nM DTX; Figure S3.3: NP distribution in HeLa treated with 50 nM DTX; Figure S4.1: MTs and NPs in untreated CAFs; Figure S4.2: Change in MTs and NP distribution in CAFs treated with 50 nM DTX; Figure S4.3: NP distribution in CAFs treated with 50 nM DTX. Figure S5: Cell Cycle Analysis 6 h DTX.
