# Supplementary material for: Docetaxel-Mediated Uptake and Retention of Gold Nanoparticles in Tumor Cells and in Cancer-Associated Fibroblasts
Source: Cancers (Basel). 2021 Jun 24;13(13):3157. doi: 10.3390/cancers13133157 (PMC8269007; doi:10.3390/cancers13133157)
Supplement: Supplementary file 1 [file cancers-13-03157-s001.zip › cancers-1232764-supplementary.pdf]

Docetaxel Mediated Uptake and Retention of Gold Nanoparticles in Tumor Cells and in Cancer Associated Fibroblasts

Abdulaziz Alhussan, Kyle Bromma , Monica Mesa Perez, Wayne Beckham, Abraham Alexander, Perry L. Howard, and Devika B. Chithrani*

Supplementary section S1: Transmission Electron Microscopy images of GNPs

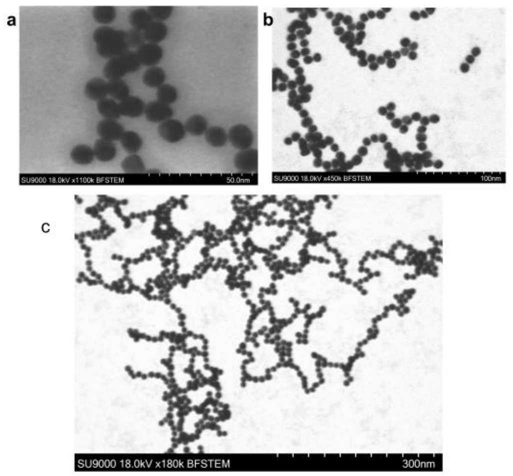

Supplementary Figure S1. a-c) TEM images of GNPs used in this study.

Supplementary section S2: UV-Visible data for functionalized GNPs

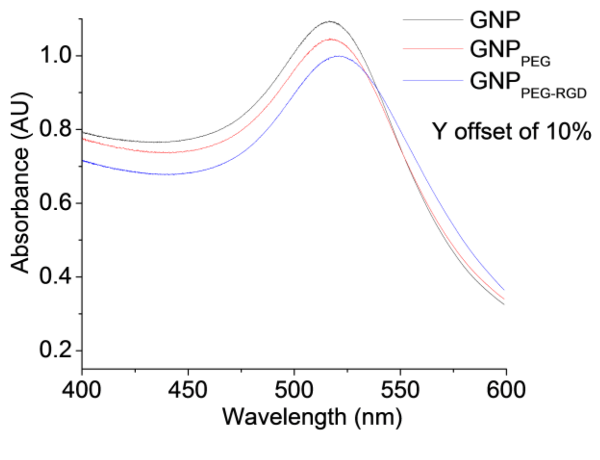

Supplementary Figure S2. UV-Visible data for functionalized NPs. A "Y-offset" of 10% was used to show the data clearly.

Supplementary section S3.1: MT network and distribution of NPs in tumor cells (HeLa)

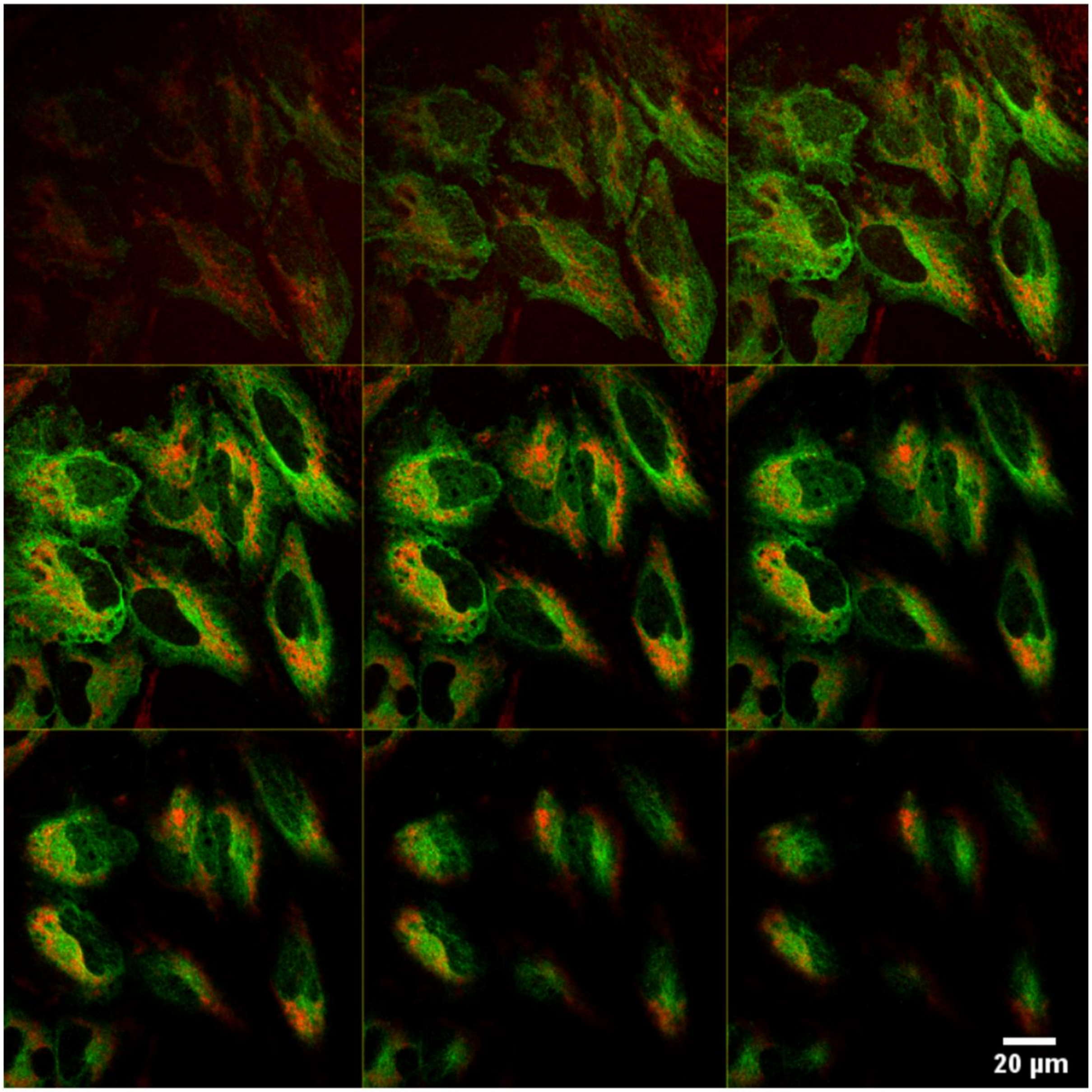

Supplementary Figure S3.1. MT network and distribution of NPs in HeLa cells across many planes of a cell. Vesicles containing GNPs and MT network are marked in red and green, respectively. Scale bar is 20 µm.

<u>Supplementary Section S3.2:</u> <u>Change in distribution of NPs in tumor cells when treated with 50 nM DTX</u>

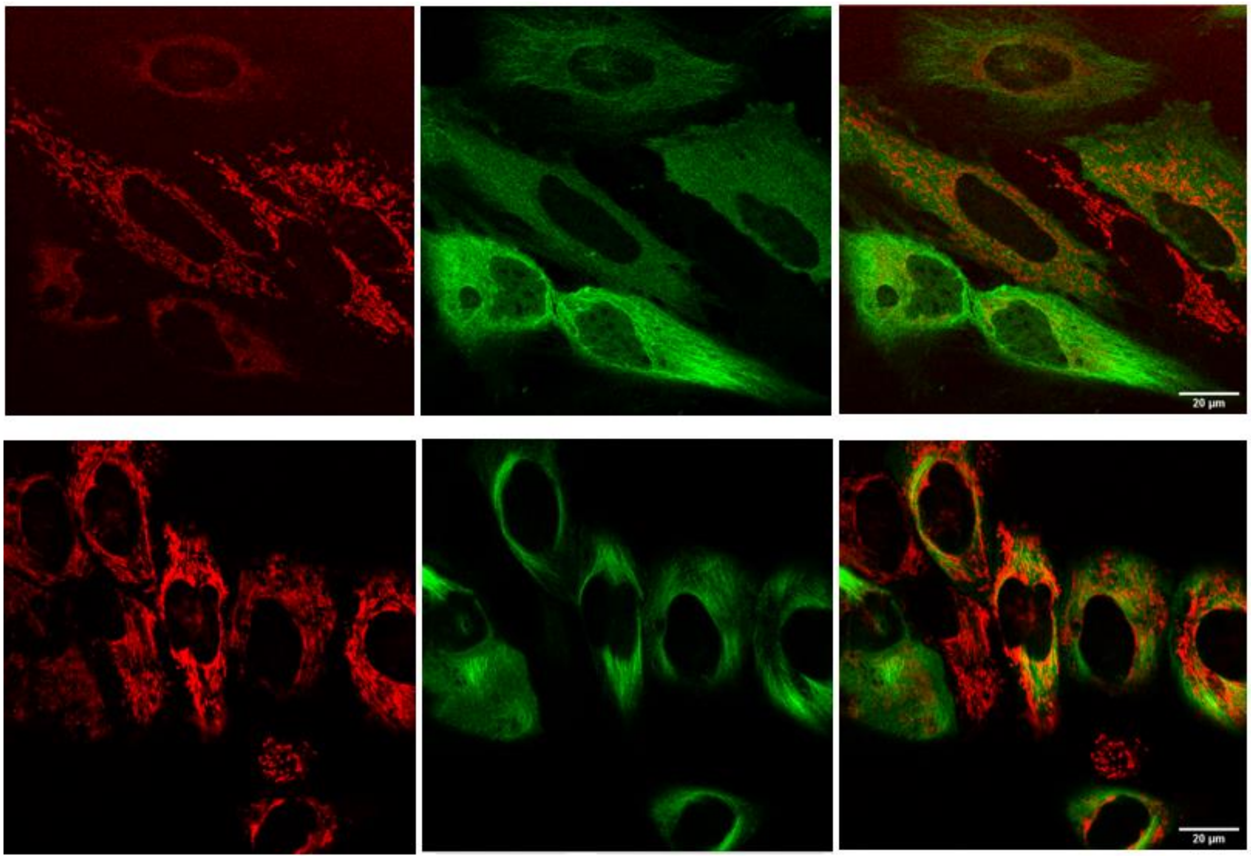

Supplementary Figure S3.2. MT network and distribution of NPs for control cells (top panel) and cells treated with 50 nM DTX (bottom panel). Scale bar is 20 µm. Vesicles containing GNPs and MT network are marked in red and green, respectively.

Supplementary Section S3.3: Distribution of NPs in tumor cells treated with 50 nM DTX

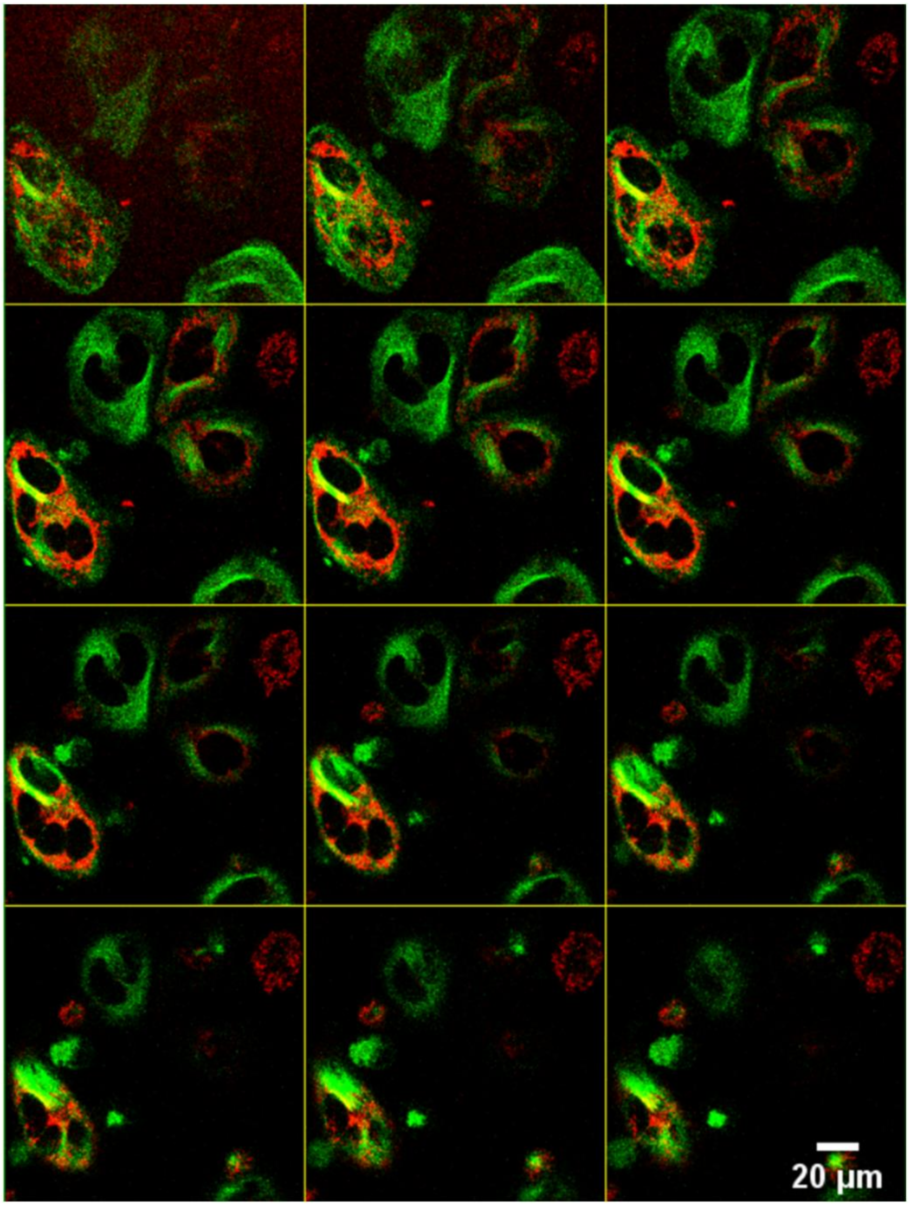

Supplementary Figure S3.3. MT network and distribution of NPs across many planes of a cell treated with 50 nM DTX. Scale bar is 20 µm. Vesicles containing GNPs and MT network are marked in red and green, respectively.

Supplementary Section S4-1: MT network and distribution of NPs in cancer associated fibroblasts (CAFs).

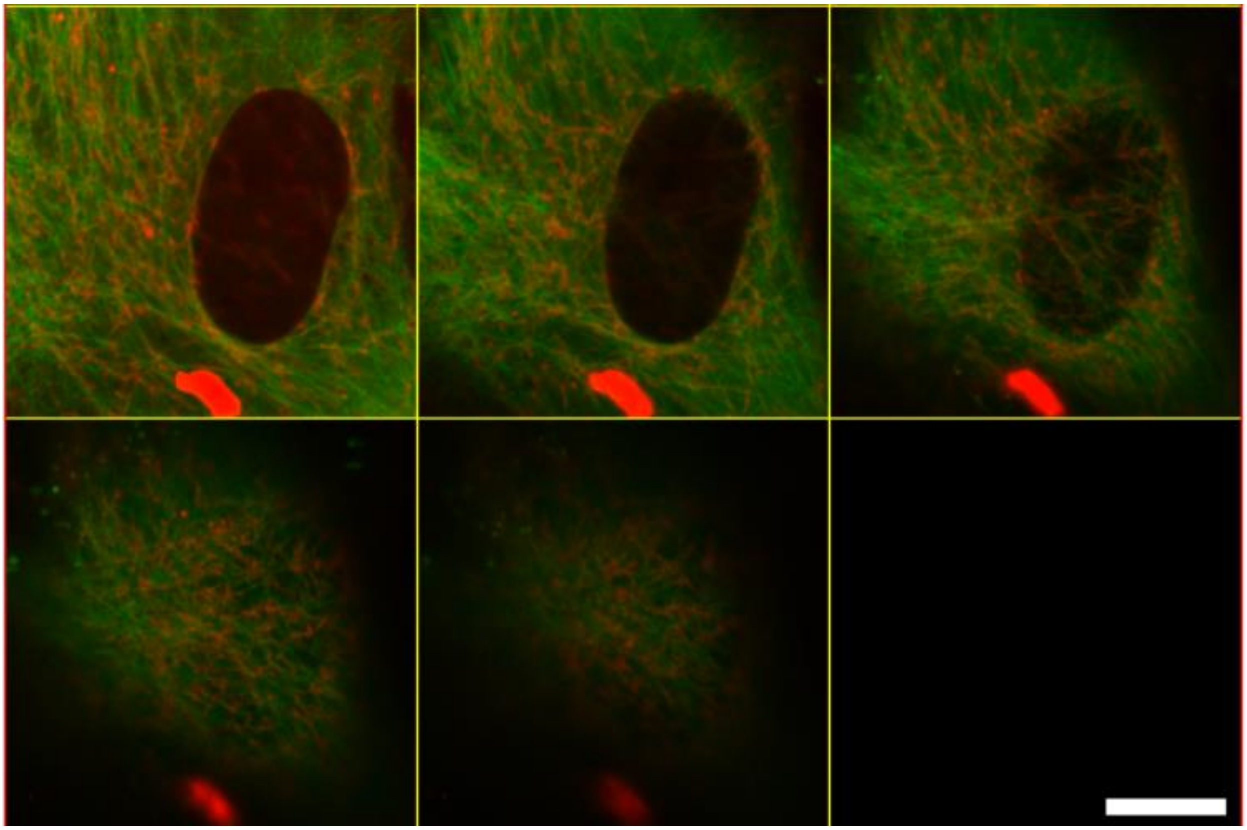

Supplementary Figure S4.1. MT network and distribution of NPs in CAFs across many planes of a cell. Scale bar is 20 µm. Vesicles containing GNPs and MT network are marked in red and green, respectively.

<u>Supplementary Section S4-2:</u> <u>Variation of distribution of NPs and MT network in cancer associated fibroblasts (CAFs) with the treatment of DTX.</u>

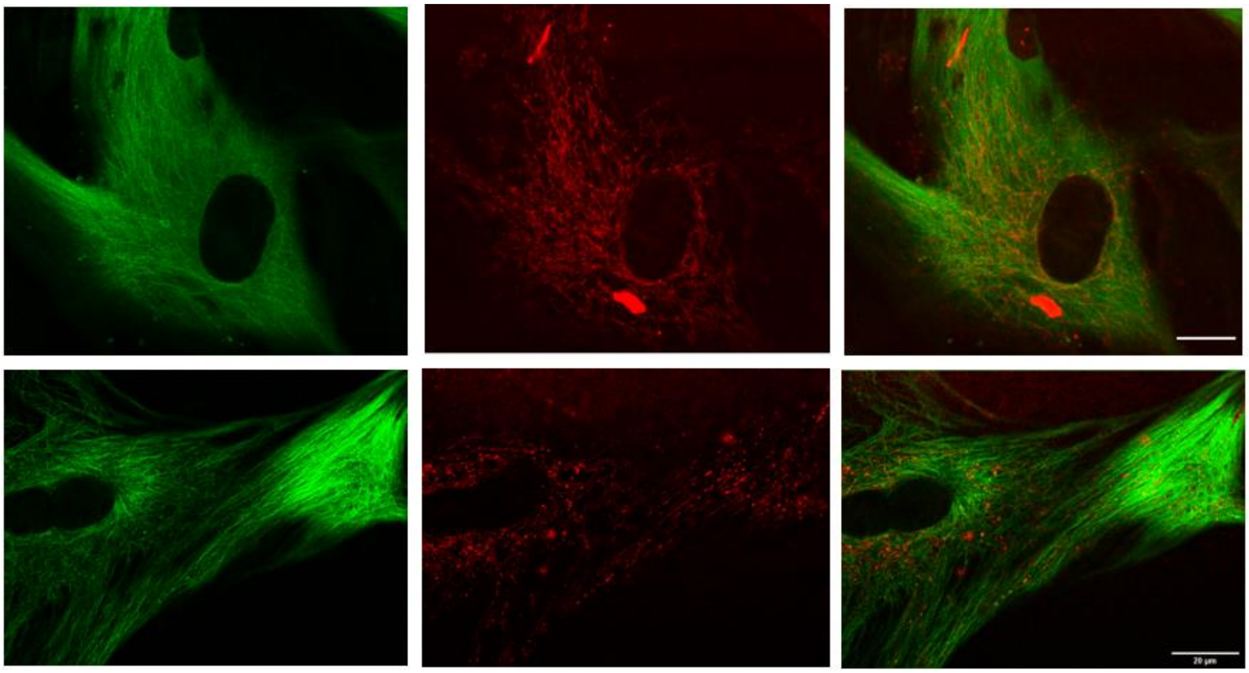

Supplementary Figure S4.2. MT network and distribution of NPs for control cells (top panel) and cells treated with 50 nM DTX (bottom panel). Scale bar is 20 µm. Vesicles containing GNPs and MT network are marked in red and green, respectively.

<u>Supplementary Section S4-3:</u> <u>Variation of distribution of NPs and MT network in cancer associated fibroblasts (CAFs) with treatment of DTX.</u>

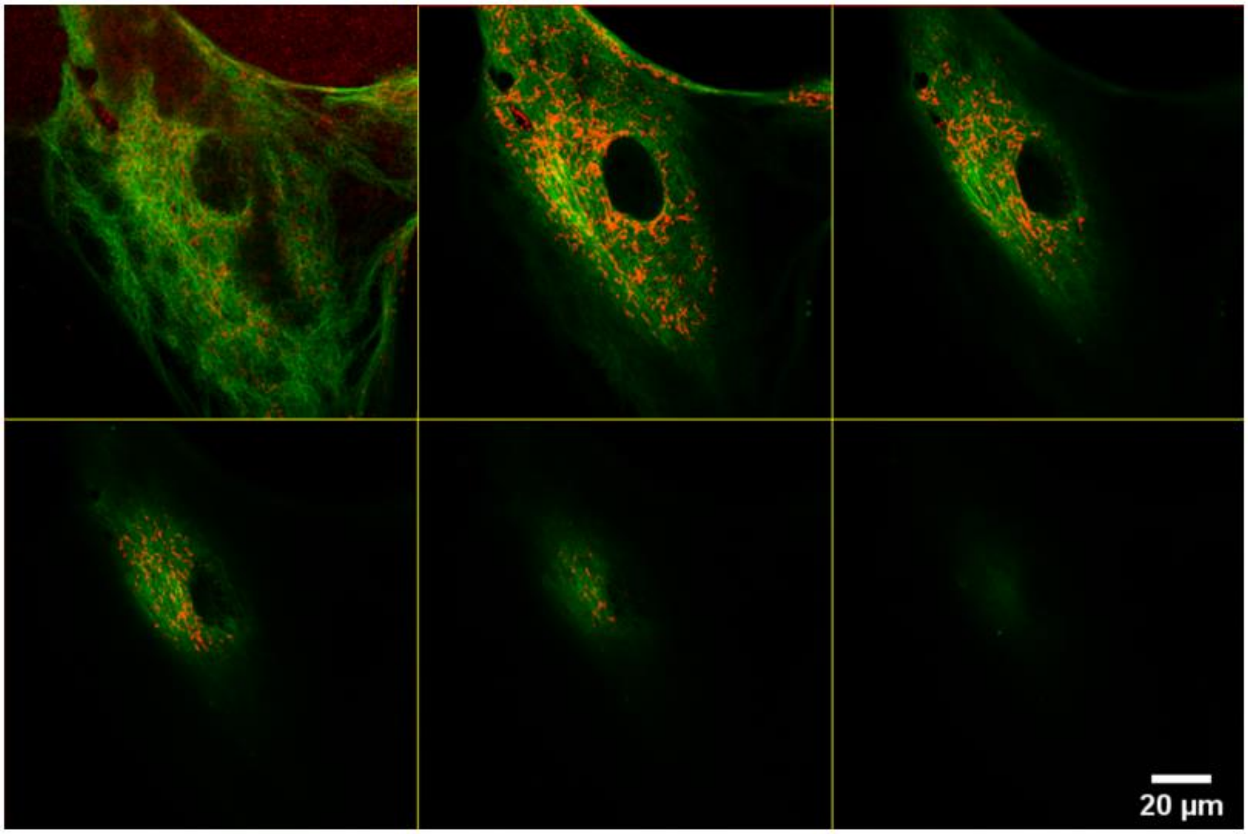

Supplementary Figure S4-3. MT network and distribution of NPs across many planes of a cell treated with 50 nM DTX. Scale bar is 20 μm. Vesicles containing GNPs and MT network are marked in red and green, respectively.

<u>Supplementary Section S-5:</u> <u>Cell Cycle Analysis.</u>

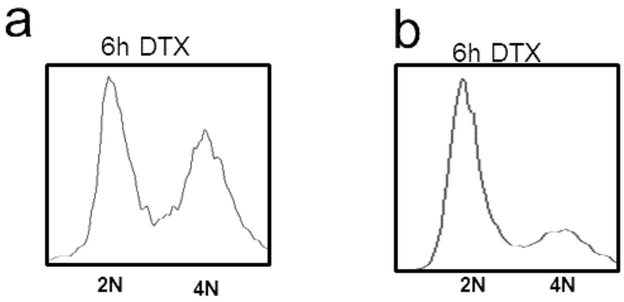

Supplementary Figure S-5. Cell Cycle Analysis of MIA PaCa-2 (a) and CAF-98 (b) 6 hours post 50 nM DTX treatment.
